# Direct comparison two fixed-ratio combination glucagon-like peptide receptor agonist and basal insulin on glycemic and non glycemic parameters in type 2 diabetes

**DOI:** 10.1186/s12902-023-01282-w

**Published:** 2023-02-01

**Authors:** Ivona Risovic, Mirjana Sumarac Dumanovic, Mirjana Bojic, Danijel Djekic

**Affiliations:** 1 Faculty of Medicine, University of Banja Luka, Banja Luka, Bosnia and Herzegovina; 2 Department of Endocrinology, University Clinical Center of the Republic of Srpska, Banja Luka, Bosnia and Herzegovina; 3 School of Medicine, University of Belgrade, Clinic for Endocrinology, Diabetes and Diseases of Metabolism, Belgrade, Serbia

**Keywords:** Fixed-ratio combination, Type 2 diabetes, Insulin degludec, Insulin glargine; liraglutide, Lixisenatide

## Abstract

**Background:**

Two types of fixed-ratio combinations of basal insulin and a glucagon-like peptide-1 receptor agonist (GLP-1RA) have been approved for use in type 2 diabetes. One is insulin degludec/liraglutide (iDergLira), and the other is insulin glargine/lixisenatide (iGlarLixi). Direct comparisons between these two combination is not available.

**Methods:**

The retrospective study included 186 patients with type 2 diabetes mellitus (DM) with inadequate glycemic control on metformin and basal insulin (degludec, glargine 100, glargine 300) who were switched to fixed-ratio combination GLP-1 RA and basal insulin. Patients were divided into two groups based on the basal insulin before study: group I (*n* = 86) treated with degludec were switched to iDegLira and patients group II (*n* = 99), treated with glargine were switched to iGlarLixi. The aim of this study was to directly compare the effects between two fixed – ratio combination on glycemic parameters and non glycemic parameters. Follow up was 6 months.

**Results:**

Mean HbA1c decreased similarly (− 1.2% vs.-1.1%). Higher percentage patients in iDegLira group had reached the HbA1c < 7% after 6 months (22% vs. 18.2%, *p* < 0.05). The mean change in fasting plasma glucose (FPG) was comparable for the two groups, while mean decrease postprandial plasma glucose (PPG) level were lower in iGlarLixi group (2 vs 1.8 mmol/l, *p* > 0.05). Change in body weight was significant in iDegLira group (1.8 kg vs. 0.7 kg, *p* < 0.001). At the end of the study patients showed decrease in total cholesterol (TC) and low-density lipoprotein (LDL) for 0.2 mmol/L in iDegLira, 0.1 mmol/l in iGlarLixi, triglycerides decreased 0.3 mmol/l in both groups, high-density lipoprotein(HDL) increased 0.1 mm/l in iGlarLixi.

**Conclusion:**

Our results showed that more patients with iDegLira had HbA1c less than 7% and these combination had better effect on weight loss. There was no difference observed in FPG and PPG, lipid profile and rate of hypoglycemia.

## Background

Diabetes mellitus (DM) type 2 is a chronic, progressive disease, resulting in multiple pathophysiologic defects including insulin resistance, reduced beta cell function, increased hepatic glucose output, inappropriate glucagon secretion, and decreased incretin effect [1, 2]. Achieving and maintaining glycemic control is essential for reducing the risk of diabetes associated microvascular complications (retinopathy, nephropathy, and neuropathy) and in many cases reducing the risk of macrovascular complications such as myocardial infarction [3].

The combination therapy with a long-acting basal insulin and a glucagon-like protein-1 receptor agonist (GLP-1RA) in patients with type 2 DM is based on the solid understanding of their complementary mechanisms of actions, which potentiate each other by acting through different mechanisms in different tissues [4]. This approach is also supported by the full clinical data. For patients already being treated with basal insulin, the addition of a GLP-1RA to their therapeutic regimen is an option when intensification therapy is needed [2, 4, 5]. This combination therapy shows the same efficacy as the basal bolus treatment regimen with respect to the glycemic control, but with a lower risk of hypoglycemia and weight gain, and is obtained with a lower dose of insulin [2, 5].

There are two dual-agent products that are available combining basal insulin with GLP-1 RA, insulin degludec/liraglutide (iDegLira) and insulin glargine/lixisenatide (iGlarLixi) [6]. Both combinations are indicated for treatment of type 2 diabetes as an adjunct to lifestyle changes including diet and exercise, and are administered subcutaneously once daily. IDegLira is a fixed-ratio combination of a once-daily long-acting basal insulin degludec and a long acting, once-daily GLP-1 RA, liraglutide. IGlarLixi is a fixed-ratio combination of a once-daily long-acting basal insulin glargine and a short-acting, once-daily GLP-1 RA, lixisenatide. There are no trials directly comparing the two fixed ratio combination GLP-1RA and basal insulin products [6–10]. The primary end point of this study was to compare the effectivness of two fixed – ratio combinations (iDegLira and iGlarLixi) on glycated hemoglobin A1c (HbA1c). The secondary end points were changes in 2 hours postprandial glucose (PPG), fasting plasma glucose (FPG)), proportion of patients achieving HbA1c < 7% and change in non glycemic parameters: body weight and lipid parameters.

## Methods

### Study design and subjects

A total of 186 patients with type 2 DM were included in this retrospective study at the University Clinical Centre of the Republic Srpska in Banja Luka. Data were collected from patient medical records. The inclusion criteria were inadequate glycemic control on metformin and basal insulin (degludec, glargine 100, glargine 300). Inadequate glycemic control was defined as HbA_1_c ≥ 7.0%. Patients had to have been treated with a basal insulin for at least 6 months before study, with a stable regimen for at least 3 months. The permitted oral antihyperglycemic drugs at screening were metformin (≥1500 mg/day or maximal tolerated dose). Patients were divided into two groups based on the basal insulin before study. Patient group I (*n* = 86, 46 man and 50 women) treated with degludec as basal insulin before study, were switched to iDegLira. Patients group II (*n* = 99, 48 man and 51 women) treated with glargine 100 or 300 < 30 units as basal insulin before study, were switched to iGlarLixi dose 20 units:10 μg. IDegLira was self –administrated once daily, at approximately the same time each day. IGlarLixi was self –administrated once daily half an hour before breakfast. The starting dose of fixed combination was determined from the last basal insulin dose received before study. Every patient got titration algorithm by physician. Exclusion criteria were use of an oral agent other than the metformin and previous discontinuation of a GLP-1 RA due to safety, tolerability. All procedures were in accordance with the ethical standards of the responsible committee on human experimentation (institutional and national) and with the Helsinki Declaration of 1964, as revised in 2013. The study protocol was approved by the Ethics Committee. All subjects gave written informed consent for participation.

### Clinical and biochemical measurements

For each patient the following demographic data were collected: gender, age, body height and weight. The following parameters were measured from baseline and after 6 months: FPG, 2 hours PPG, HbA1c, total cholesterol (TC), high -density cholesterol (HDL), low- density cholesterol (LDL), triglycerides. Hypoglycemia defined as glycemia less than 3.9 mmol/l. Hypoglycemia was recorded based on the information from self monitored glucose levels in the medical records.

### Statistical analysis

Central tendencies, mesures of variability and relative numbers were used for descriptive statistical methods. The normality of the distribution of continuous data was examined by graphical and mathematical methods. To compare means between continuous variables between two groups we used T test (or Mann Whitney test), depending of data distribution. To comapare change from baseline to 6 months we used T tets for continuos variables. Mc Nemar test was uses to comapare change from baseline to 6 months for nominal data. Correlation between the observed variables was assessed using the Spearman or Person correlation. A *p*-value < 0.05 was considered statistically significant. All analyses were performed using the SPSS Statistics.

## Results

There were no difference in baseline and demographic characteristics between the treatments groups, Table 1. Patients were mean age of 60 years, were generally obese and had a mean duration of diabetes 10 years, Table 1.

**Table 1:** Baseline, demographics and disease characteristics, baseline and the end of study

	IDegLira			IGlarLixi		
	Baseline	At the end	*p* value	Baseline	At the end	*p* value
Gender,male, %	53.5	53.5	ns	48.5	48.5	ns
Age, years	58.6 ± 9.7	59.2 ± 3.5	ns	60.2 ± 8.3	60.8 ± 7.4	ns
Duration of DM, years	9.6 ± 3.6	10.2 ± 3.2	ns	10.5 ± 3.9	11.1 ± 4.1	ns
HbA1c, %	8.9 ± 1.1	7.7 ± 1	< 0.001	9 ± 1.4	7.9 ± 1.1	< 0.001
FPG, mmol/l	7.5 ± 1.5	6.0 ± 1.1	< 0.001	7.8 ± 1.7	6.4 ± 0.37	< 0.001
PPG, mmol/l	10.1 ± 2.5	8.3 ± 0.9	< 0.001	10.3 ± 1.8	8.3 ± 1.2	< 0.001
Weight, kg	87.1 ± 15.4	85.3 ± 14.5	< 0.001	89.4 ± 14.9	88.7 ± 14.5	< 0.001
BMI, kg/m^2^	30.2 ± 7.3	28.8 ± 3.2	< 0.001	30.8 ± 4.3	30.2 ± 4.1	< 0.001
Insulin dose, units	27 ± 5.5	26 ± 4	< 0.05	28 ± 5	26 ± 3.5	< 0.001

Data are presented as % or mean ± SD. HbA1c-glycated hemoglobin A1c, FPG-fasting plasma glucose, PPG- postprandial glucose, DM-diabetes mellitus, BMI-body mass index, not significant.

**Fig. 1:**
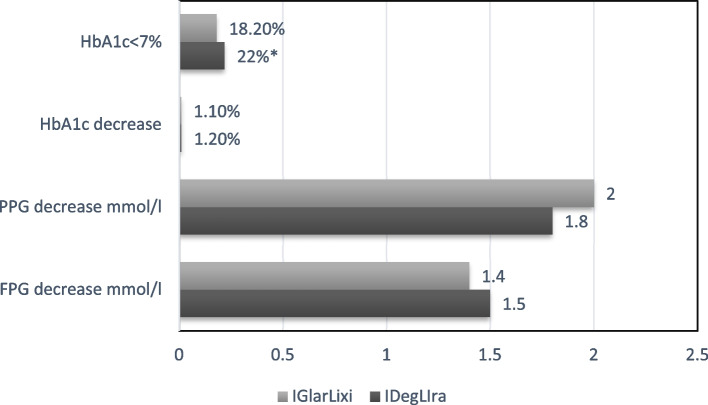
Mean change in glycemic parameters

Mean HbA1c decreased similarly in two treatments groups (−1.2% vs.-1.1%), Fig. 1. There were statistical differences in the change in HbA1c from baseline to 6 months in both groups, Table 1. Higher percentage patients in iDegLira group had reached the HBA1c < 7% after 6 months (22% vs. 18.2%, *p* < 0.05), Fig. 1. The mean change in FPG was comparable for the two groups, while mean decrease PPG level was lower in iGlarLixi group, but these difference no significance (2 vs 1.8 mmol/l, *p* > 0.05), Fig. 1.

**Fig. 2:**
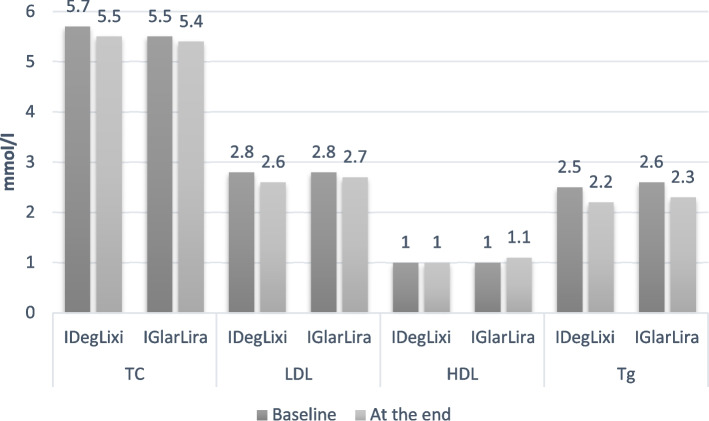
Mean change in lipid parameters

At the end of the study patients showed decrease in TC and LDL for 0.2 mmol/L in iDeg Lira and 0.1 mmol/l in iGlar Lixi, triglycerides for 0.3 mmol/l in both groups, increase HDL for 0.1 mm/l in iGlarLixi, Fig. 2

Changes in body weight were significant difference of 1.8 kg from baseline in iDegLira group, than 0.7 kg in iGlarLixi group (*p* < 0.001), Table 1. After 6 months in both groups were significantly less hypoglycemia episodes per week. In iDegLira group without hypoglycemia were 97.7%, in iGlarLixi 96%, with 1–2 hypoglycemia per week in iDegLira 2.3% and in iGlarLixi 3%. At the end of study there were no more than 2 hypoglycemia episodes per week in each group. There were not any participant drop outs during the study.

## Discussion

Diabetes impacts multiple systems within the body, and achievement and maintenance of glycemic goals are important in preventing or at least delaying the development and progression of diabetes-associated complications [11]. To effectively treat type 2 diabetes, treatment intesification is often required using combinations of medications that address one or the more of the many pathologic processes associated with the disease [4, 5, 12]. The fixed-ratio combination therapy is appropriate for patients who are already taking either a basal insulin or a GLP-1 RA but still show insufficient glycemic control [13]. Patients who may particularly benefit from such a therapy include those who want to avoid the multiple injections required with prandial insulin in an insulin intensification regimen, as well as the frequent blood glucose testing needed to adjust prandial doses and lessen the risk of hyper/hypoglycemia [4, 5, 12, 13]. Combination therapy using the complementary mechanisms of action of basal insulin and GLP-1 RA targets seven of the many pathophysiologic defects in type 2 diabetes, adressing both FPG and PPG [5, 8, 12]. No head-to-head comparison has been done with fixed-ratio combinations and therefore it is not possible to make recommendations between the two for glycemic control alone [13–15].

With a mean diabetes duration od 10 years, mean BMI of 30 kg/m^2^ and already on two glucose lowering agens, population of this study represents a group of patients that is challenging to treat successfully in the clinical setting. These patients need more than one glucose-lowering drugs to maintain glycemic control.

Our results had showed higher proportions of patients with iDegLira reaching the HbA1c < 7% and greater reductions in HbA1c. Other studies had showed higher reduction HbA1c than our results [15, 16]. A systematic review and meta-analysis of trials indirectly compared iGlarLixi and iDegLira and showed the mean change in HbA1c was 1.50% after iGlarLixi treatment and 1.89% after iDegLira treatment. Evans et al. showed in indirect comparison the mean reduction in HbA1c was 0.44% greater with IDegLira compared to iGlarLixi, and a greater proportion of patients reached HbA_1c_ < 7.0%. The results of this indirect treatment comparison demonstrate that, among patients with type 2 diabetes uncontrolled on basal insulin, treatment with IDegLira resulted in a greater reduction of HbA1c and higher odds of reaching HbA1c < 7% compared with iGlarLixi [14]. Other meta analises showed similar results [11, 15, 16]. We didn’t find difference between iDegLira and iGlarLixi for change in FPG. Groups with iGlarLixi had marked decrease in PPG. Other meta analyses had showed favour od iDegLira for change in FPG, but iGlarLixi for PPG. IGlarLixi contain short acting GLP-1 RA with a predominant PPG lowering effect [5, 6, 16–18].

Better glycemic control did not associated with higher rate of hypoglycemia. We did not found significant difference in hypoglycemic episodes between groups. AT the end of study the number of hypoglycemia episodes was reduced in both groups. A lower incidence of hypoglycemia indicating that the fixed ratio combination may mitigate the increased risk of hypoglycemia often seen in long-standing DM. Other studies also showed good safety profile of fixed ratio combination therapy [12, 13].

Obesity in major risk factors for type 2 diabetes. Modest weight loss can minimize and reduce diabetes-associated complications [19]. In our study fixed combination iDegLira was superior in body weight and BMI reduction than iGlarLixi. Different GLP-1 RA have different effect on weight change. Differences in uptake across the blood–brain barrier (or in brain access through subfornical organs) have been postulated as an explanation [20–23]. Clinical trial data from patients receiving fixed-ratio combination showed a weight change of − 0,3 to − 0,7 kg for iGlarLixi and − 2 to − 2,7 kg for iDegLira [12, 13, 16, 17, 24].

Our results had showed improvements in lipid levels. Both preparation had showed the most effect on triglycerides level. Although, iDegLira more decrease TC and LDL, these differences were not significant. Other studies showed better lipid profil with fixed ratio combination [25–30]. Unlike other results which did not show diffrence in HDL level, our results alsoshowed increase in HDL level in iGlarLixi groups.

These retrospective design had some limitations, so potential confounding factors cannot be ruled out. Participants in clinical trials have more careful follow-up, titration guidelines are more aggressively enforced than in usually done in routine clinical practice. Therefore, further studies, like RCTs would be desirable.

## Conclusion

Our results had showed that more patients with iDegLira had HbA1c less than 7% and these combination had better effect on weight loss. Both preparations had similar effect on FPG and PPG, benefit on lipid profile and similar rate of hypoglycemia.

## Data Availability

Original data sets and materials are not publicly available because of patient privacy protection but are available from the corresponding author upon reasonable request.

## Abbreviations

DM: Diabetes mellitus
GLP-1RA: Glucagon-like protein-1 receptor agonist
iDegLira: Insulin degludec/liraglutide
iGlarLixi: Insulin glargine/lixisenatide
BMI: Body mass index
HbA1c: Glycated haemoglobin A1c
FPG: Fasting plasma glucose
PPG: Postprandial glucose
TC: Total cholesterol
HDL: High density cholesterol
LDL: Low density cholesterol.
